# Dehydrozingerone exerts beneficial metabolic effects in high-fat diet-induced obese mice *via*AMPK activation in skeletal muscle

**DOI:** 10.1111/jcmm.12455

**Published:** 2015-01-12

**Authors:** Su Jin Kim, Hong Min Kim, Eun Soo Lee, Nami Kim, Jung Ok Lee, Hye Jeong Lee, Na Yeon Park, Joo Yeon Jo, Bo Young Ham, Si Hyun Han, Sun Hwa Park, Choon Hee Chung, Hyeon Soo Kim

**Affiliations:** a Department of Anatomy, Korea University College of MedicineSeoul, South Korea; b Department of Internal Medicine, Yonsei University Wonju College of MedicineWonju, South Korea; c Seoul Science High SchoolSeoul, South Korea

**Keywords:** AMPK, curcumin analogue, dehydrozingerone, glucose uptake, metabolism

## Abstract

Dehydrozingerone (DHZ) exerts beneficial effects on human health; however, its mechanism of action remains unclear. Here, we found that DHZ suppressed high-fat diet-induced weight gain, lipid accumulation and hyperglycaemia in C57BL/6 mice and increased AMP-activated protein kinase (AMPK) phosphorylation and stimulated glucose uptake in C2C12 skeletal muscle cells. DHZ activated p38 mitogen-activated protein kinase (MAPK) signalling in an AMPK-dependent manner. Inhibiting AMPK or p38 MAPK blocked DHZ-induced glucose uptake. DHZ increased GLUT4 (major transporter for glucose uptake) expression in skeletal muscle. Glucose clearance and insulin-induced glucose uptake increased in DHZ-fed animals, suggesting that DHZ increases systemic insulin sensitivity *in vivo*. Thus, the beneficial health effects of DHZ could possibly be explained by its ability to activate the AMPK pathway in skeletal muscle.

## Introduction

Dehydrozingerone (DHZ), isolated from ginger (*Zingiber officinale*), is an unsaturated derivative of the natural product zingerone. DHZ exerts anti-oxidant 1,2, free radical scavenging 3, and anti-tumour activities 4,5, and also inhibits wound healing 6 and lipid peroxidation 7. Metabolic roles for DHZ have been proposed, but the molecular mechanisms underlying these effects remain unclear.

AMP-activated protein kinase (AMPK) is an evolutionarily conserved sensor of the cellular energy status 8,9. AMPK is activated in response to increases in the cellular AMP:ATP ratio, which results in the acceleration of ATP-generating pathways and the inhibition of ATP-consuming pathways 10,11. AMPK can be activated by multiple regulatory pathways 12. Although the molecular mechanisms underlying AMPK activation have yet to be elucidated thoroughly, activation requires the phosphorylation of the catalytic α-subunit on Thr-172 within the activation loop 13,14. Both LKB1 and calcium/calmodulin-dependent protein kinase (CaMKK) have also been associated with AMPK 15,16. Upon activation, AMPK causes increases in glucose uptake, fatty acid oxidation and mitochondrial biogenesis.

Curcumin, also known as diferuloylmethane, is a hydrophobic polyphenol derived from the rhizome of the herb *Curcuma longa*. It exerts anti-oxidant 17, anti-inflammatory 18, anti-tumour 19, anti-diabetic 20 and neuroprotective 21 effects. We previously found that curcumin stimulates glucose uptake in skeletal muscle cells 22. Although curcumin may be useful clinically, it has not yet been approved as a therapeutic agent. The low bioavailability of curcumin may limit its clinical potential. DHZ is a half analogue of curcumin, representing half of its chemical structure. Numerous studies have suggested that curcumin analogues are promising phytochemicals for the treatment of metabolic diseases such as diabetes and obesity. However, only a few studies have investigated the effects of curcumin analogues in metabolic diseases.

We investigated the effects of DHZ on metabolic profiles to characterize its metabolic effects. In this study, we determined that DHZ suppressed high-fat diet (HFD)-induced increases in glucose and cholesterol *via* a mechanism involving AMPK.

## Materials and methods

### Reagents

Glucose, insulin, curcumin, and 1, 7-bis (4-hydroxy-3-methoxyphenyl)-1, 6-heptadiene-3, 5-dione (DHZ) were purchased from Sigma-Aldrich (St. Louis, MO, USA). 5-Aminoimidazole-4-carboxamide-1-β-ribofuranoside (AICAR) was purchased from Toronto Research Chemical Incorporation (Toronto, ON, Canada). DHZ and SB203580 [a p38 mitogen-activated protein kinase (MAPK) inhibitor] were obtained from BIOMOL International LP (Butler Pike, PA, USA). Polyclonal anti-phospho AMPKα, p38 MAPK, insulin receptor substrate-1 (IRS-1), Akt and inactive AMPKα, p38 MAPK, IRS-1, Akt and anti-β-actin antibodies were purchased from Millipore Millipore, (Billerica, MA, USA) (MA, USA). GLUT4 antibody was purchased from Abcam (Cambridge, UK). Compound C, an AMPK inhibitor, was provided by Merck (RY 70-100; Rahway, NJ, USA). Hybond ECL nitrocellulose membrane was obtained from Amersham (Arlington Heights, IL, USA). All cell culture reagents and other chemicals were purchased from Life Technologies (Gaithersburg, MD, USA).

### Cell culture

Mouse C2C12 myoblasts and L6 rat skeletal muscle cells were maintained in α-MEM (Sigma-Aldrich) supplemented with 100 mg/ml kanamycin and 10% foetal bovine serum at 37°C in 100-mm cell culture dishes, under a humidified atmosphere containing 5% CO_2_.

### RT-PCR

First strand cDNA synthesis was performed with 1 μg of total RNA isolated from C2C12 cells at 55°C for 20 min. using the Thermoscript II one-step RT-PCR Kit (Life Technologies, Paisley, UK). cDNA amplification was performed in the same tube using the Gene Amp System 9700 thermocycler (Applied Biosystems, Warrington, UK) followed by heating to 94°C for 5 min. to inactivate the reverse transcriptase. The following PCR conditions were used: 34 cycles each of 30 sec. at 94°C, 30 sec. at 55°C and 60 sec. at 72°C, followed by 10 min. at 72°C. The number of PCR cycles used was optimized to ensure amplification at the exponential phase. Ten-microlitre samples from each RT-PCR reaction were removed and analysed by agarose gel electrophoresis. Bands were stained with ethidium bromide and visualized under ultraviolet (UV) light. The band intensities were quantified using a gel documentation system (Gene Genius, Syngene, UK). The following primers were used: GLUT4-sense (5′-TTG GAG AGA GAG CGT CCA AT-3′) and GLUT4-antisense (5′-CTC AAA GAA GGC CAC AAA GC-3′); β-actin-sense (5′-CAG GAG GAG CAA TGA TCT TGA-3′) and β-actin antisense (5′-ACT ACC TCA TGA AGA TCC TCA-3′). RT-PCR experiment with animal tissues was also performed. Various primers were used as indicated.

### Western blotting

C2C12 cells were grown in 6-well plates until 60–70% confluency, serum starved for 24 hrs, and then treated at 37°C, as indicated. The media were aspirated, and the cells were washed twice in ice-cold PBS and then lysed in 100 μl lysis buffer [0.5% deoxycholate, 0.1% SDS, 1% Nonidet P-40, 150 mM NaCl and 50 mM Tris-HCl (pH 8.0)] containing proteinase inhibitors (0.5 μM aprotinin, 1 μM phenylmethylsulfonyl fluoride and 1 μM leupeptin) (Sigma-Aldrich). The supernatants were sonicated briefly, heated for 5 min. at 95°C, centrifuged for 5 min., separated on SDS-PAGE (8–16%) gels, and transferred to nitrocellulose membranes. The blots were then incubated overnight at 4°C with primary antibodies and washed six times in Tris-buffered saline/0.1% Tween 20, before 1-hr incubation with horseradish peroxidase-conjugated secondary antibodies at room temperature. The blots were also incubated with anti-β-actin antibodies to normalize protein loading. All blots were visualized using ECL (Amersham Biosciences, Buckinghamshire, UK). The membrane was scanned and densitometry analysis was performed with an Image J analysis.

### 2-Deoxyglucose uptake

The uptake of 2-deoxyglucose by L6 cells was evaluated. Briefly, cells were rinsed twice with warm PBS (37°C), and then starved in serum-free DMEM for 3 hrs. After treatment, the cells were incubated in KRH buffer (20 mM HEPES, pH 7.4, 130 mM NaCl, 1.4 mM KCl, 1 mM CaCl_2_, 1.2 mM MgSO_4_, 1.2 mM KH2PO4) containing 0.5 μCi of 2-deoxy-D [H^3^] glucose for 15 min. at 37°C. The reaction was terminated by placing the plates on ice and washing twice with ice-cold PBS. The cells were then lysed in 50 mM NaOH, and radioactivity was evaluated by scintillation counting of the SDS-extracted lysates.

### AMPKα2 silencing

C2C12 cells were seeded in 6-well plates and grown to 70% confluence for 24 hrs. Transient transfections were performed with Lipofectamine 2000 (Life Technologies) following the manufacturer's instructions. Briefly, AMPKα2 siRNA was purchased from Dharmacon (L-040809-00-0005; Thermo Scientific, Rockford, IL, USA), and non-targeted control siRNA was designed and synthesized (Bioneer, Daejon, Korea). Five microlitres of siRNA and 5 μl Lipofectamine 2000 were diluted with 95 μl reduced serum medium (Opti-MEM; Life Technologies) and mixed. The transfection mixture was incubated for 30 min. at room temperature, and then added drop-wise to each well containing 800 μl of Opti-MEM (final siRNA concentration, 100 nM). Four hours after transfection, the media were changed to fresh complete medium.

### Experimental animals

Four-week-old male C57BL/6 mice were purchased from Dae Han Bio Link Co. Ltd. (Chungbuk, Korea). They were housed in cages and placed in a room with a 12:12 hr light–dark cycle and ambient temperature. The mice were fed a commercial chow diet for 4 weeks after arrival for adaptation. At 8 weeks of age, they were divided randomly into three groups with 10 animals per group. The groups were then fed an HFD, an HFD with DHZ, or a normal diet for 12 weeks. Food intake and bodyweights were recorded weekly. After 12 weeks, animals were anaesthetized with Zoletil® (Virvac Laboratories, Carros, France) by intraperitoneal injection. Blood samples were harvested by cardiac puncture into tubes containing heparin solution and centrifuged at 2000 × g for 10 min. to obtain plasma. The plasma was then stored at −80°C until use. Mice were perfused with 0.9% saline, and tissues were removed and stored for the analysis of mRNA expression or fixed with 4% paraformaldehyde for histological examination.

### Glucose tolerance test

All experimental animals were fasted for ∼16 hrs before 20% d-dextrose (2 g/kg) was injected intraperitoneally. The blood glucose levels were measured before and at 15, 30, 60 and 120 min. after injection.

### Plasma analysis

The plasma levels of glucose (Asan Pharm Co., Seoul, Korea), insulin (Shibayagi Co., Shibukawa, Japan), leptin, adiponectin (both AdipoGen Inc., Seoul, Korea), and total cholesterol (Asan Pharm Co.) were measured using commercial ELISA kits following the manufacturer's instructions.

### Statistical analysis

One-way anova and Holm–Sidak comparisons were used to compare insulin secretion between groups, and post hoc Fisher's test was used to compare insulin secretion. The means were considered to be statistically different when the probability of the event was determined to be below 5% (*P* < 0.05). In addition, the data from animal experiments were analysed by anova using the Statistical Analysis System. Significant differences between groups were determined using Student's *t*-test or Duncan's multiple range-test at *P* < 0.05 (* and ** respectively).

## Results

### DHZ suppresses HFD-induced increase in bodyweight

**Fig 1:**
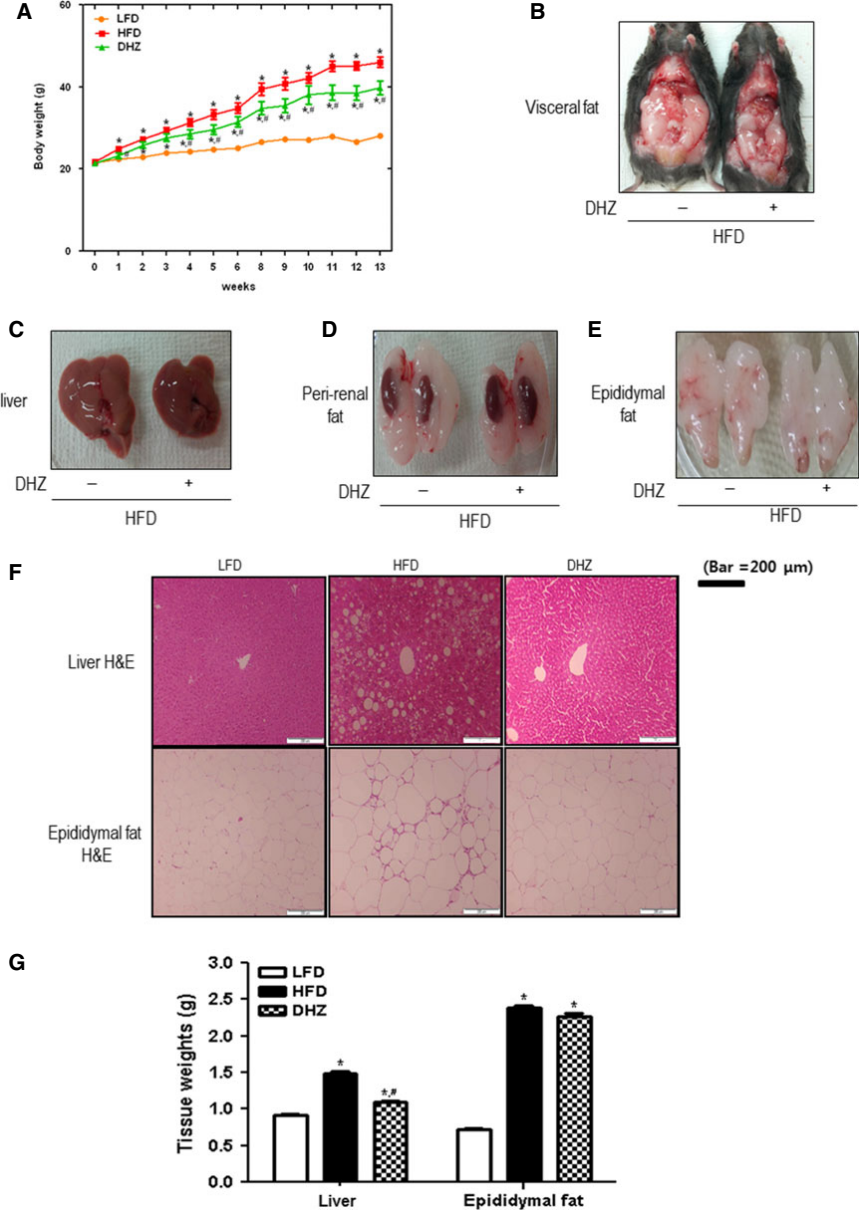
DHZ suppressed high-fat diet (HFD)-induced bodyweight gain. (A) Effects of DHZ on bodyweight in an HFD-induced obesity model. Data are provided in terms of means ± SD (*n* + 5). * Denotes significant differences compared with normal diet-fed mice (*P* < 0.05); *^, #^ denotes significant differences compared with HFD-fed mice (*P* < 0.05). (B) Effects of DHZ on visceral fat in an HFD-induced obesity model. (C) Effects of DHZ on fatty liver in an HFD-induced obesity model. (D) Effects of DHZ on the kidney and peri-renal fat in an HFD-induced obesity model. (E) Effects of DHZ on the epididymal fat in an HFD-induced obesity model. (F) Effects of DHZ on the size of adipocyte of an HFD-induced obesity model. (G) Effects of DHZ on the weight of liver and epididymal fat tissues of an HFD-induced obesity model. The three groups were as follows: LFD, low-fat diet; HFD, high-fat diet and HFD supplemented with 100 mg/kg/day DHZ.

To determine whether DHZ exerts metabolic effects in mice, we evaluated its effects on HFD-induced changes in bodyweight. The administration of DHZ suppressed HFD-induced increase in bodyweight (Fig.1A) and significantly decreased total visceral fat compared with that seen with HFD alone (Fig.1B). HFD-stimulated fatty liver was suppressed by treatment with DHZ (Fig.1C). The amount of peri-renal and epididymal fat tissue was also significantly lower in DHZ-fed mice than in HFD-fed mice (Fig.1D and E). Haematoxylin and eosin staining showed that the size of adipocyte of liver and epididymal fat was smaller in DHZ-fed mice (Fig.1F). The weight gain of tissue, such as liver, was attenuated by DHZ administration in high-fat feeding animals (Fig.1G). Together, these data suggest that DHZ reduced HFD-induced visceral fat accumulation and weight gain.

### DHZ attenuates HFD-induced increase in blood glucose

**Fig 2:**
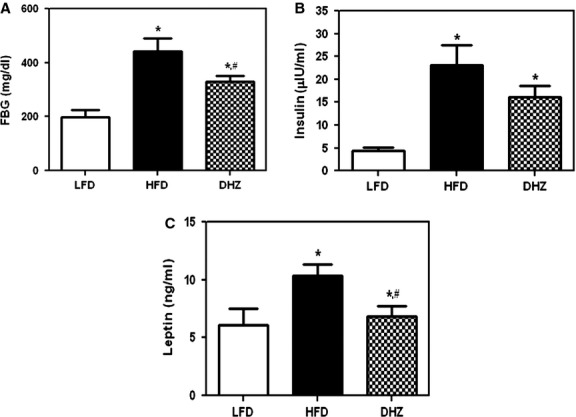
DHZ attenuates high-fat diet (HFD)-induced increase in blood glucose. (A) Effects of DHZ on HFD-induced fasting blood glucose (FBG) levels. (B) Effects of DHZ on insulin in an HFD-induced obesity model. (C) Effects of DHZ on leptin in an HFD-induced obesity model. The three groups were as follows: LFD, low-fat diet; HFD, high-fat diet and HFD supplemented with 100 mg/kg/day DHZ. Data are presented in terms of mean ± SD (*n* + 5).* Denotes significant differences compared with LFD-fed mice (*P* < 0.05); *^, #^ denotes significant differences compared with HFD-fed mice (*P* < 0.05).

We measured blood glucose levels in the experimental animals to determine whether DHZ exerted effects on glucose levels. Treatment with DHZ suppressed the HFD-induced increase in glucose and insulin levels (Fig.2A and B). The levels of leptin were also lower in DHZ-fed mice than in mice fed the HFD alone (Fig.2C). Therefore, the typical characteristics of HFD-induced type 2 diabetes were suppressed by DHZ in HFD-fed mice.

### DHZ activates AMPK phosphorylation in C2C12 cells

**Fig 3:**
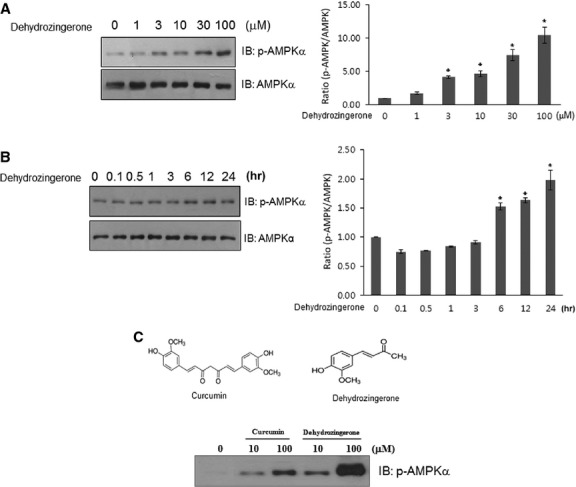
DHZ activates AMPK phosphorylation in C2C12 skeletal muscle cells. (A) C2C12 cells were stimulated for 1 hr with various concentrations of DHZ. The cells were then lysed with 2× SDS sample buffer, and the phosphorylation of AMPK was assessed by western blotting using phosphorylation-specific antibody. The level of total AMPK was also assessed as a control for protein loading. The results are representative of four independent experiments. *P < 0.05 *versus* basal condition. (B) C2C12 cells were treated with 30 μM DHZ for the indicated times. The cells were lysed with 2× SDS sample buffer, and the phosphorylation of AMPK was evaluated by western blotting using phosphorylation-specific antibody. The level of total AMPK was also assessed as a control for protein loading. The results are representative of four independent experiments. *P < 0.05 *versus* basal condition. (C) C2C12 cells were treated with DHZ and curcumin for the indicated doses. The cells were lysed with 2× SDS sample buffer, and the phosphorylation of AMPK was evaluated by western blotting using phosphorylation-specific antibody.

To assess the mechanism behind the metabolic effects of DHZ in C2C12 myoblasts, we assessed the activation of AMPK, a key regulator of glucose. The administration of DHZ induced a dose- and time-dependent increase in AMPK phosphorylation (Fig.3A and B). Maximal AMPK activation was observed with a concentration of 30 μM DHZ for 10 min. Comparing with the potency in AMPK phosphorylation, DHZ, a half analogue of curcumin, showed a stronger activity for AMPK phosphorylation than curcumin (Fig.3C). Together, these results suggest that DHZ increases the phosphorylation of AMPK in skeletal muscle and may be more promising agent than curcumin.

### DHZ stimulates glucose uptake in rat L6 myotubes

**Fig 4:**
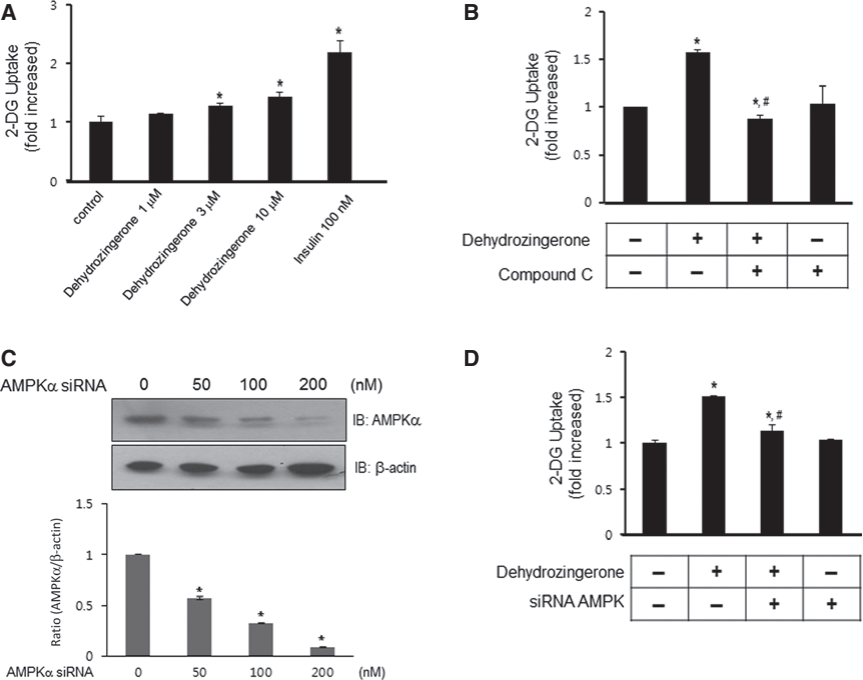
DHZ stimulates glucose uptake in rat L6 myotubes. (A) L6 myotubes were incubated in 12-well plates for 1 hr with either DHZ (1, 3, 10, 30 μM) or insulin (100 nM) alone or in combination. 2-DOG uptake was then assayed, as described in Materials and Methods. *P < 0.05, compared with control (one-way anova and Holm–Sidak comparisons). Each value is expressed as mean ± SD of four independent experiments. (B) L6 myotubes were treated for 1 hr with 10 μM DHZ in the presence of compound C (2 μM). 2-DOG uptake was then assayed, as described in Materials and methods. *P < 0.05, compared with control (one-way anova and Holm–Sidak comparisons); *^, #^ *P* < 0.05, compared with DHZ-treated cells (one-way anova and Holm–Sidak comparisons). Each value is expressed as mean ± SD of four independent experiments. (C) L6 myotubes were transiently transfected with various concentrations of siRNA against AMPKα2 for 48 hrs. The cells were lysed with 2× SDS sample buffer, and the expression of AMPKα was evaluated by western blotting. The levels of β-actin were also measured as a control for protein loading. The results are representative of four independent experiments. *P < 0.05 *versus* basal condition. (D) L6 myotubes were transiently transfected with 100 nM AMPKα2 siRNA for 48 hrs. The cells were then stimulated with 10 μM DHZ for 1 hr, and 2-DOG uptake was assayed. *P < 0.05, compared with control (one-way anova and Holm–Sidak comparisons); *^, #^*P* < 0.05, compared with DHZ-treated cells (one-way anova and Holm–Sidak comparisons). Each value is expressed as the mean ± SD of four independent experiments.

We next assessed the effects of DHZ on glucose uptake to investigate its role in skeletal muscle. L6 myotubes take up more glucose than C2C12 cells 23, suggesting that L6 cells are a better model for investigating glucose uptake. Therefore, we used L6 rather than C2C12 cells in these studies. DHZ began to increase 2-deoxyglucose uptake at a concentration of 3 μM, with the maximal effect observed with 10 μM (Fig.4A). Insulin (100 nM), used as a positive control, also caused an increase in glucose uptake. Pre-treatment with 2 μM compound C, an AMPK inhibitor, blocked DHZ-induced glucose uptake (Fig.4B), suggesting that AMPK plays a role in DHZ-induced glucose uptake. To confirm that the effects of DHZ were mediated by AMPK activation, we investigated the effects of AMPKα2 knockdown on glucose uptake by transient transfection with AMPKα2 siRNA. AMPK knockdown was confirmed by Western blotting with an anti-AMPKα antibody (Fig.4C). DHZ-induced glucose uptake decreased significantly in cells transfected with AMPKα2 siRNA (Fig.4D). Together, our findings suggest that AMPK plays an important role in DHZ-induced glucose uptake.

### DHZ activates the p38 MAPK pathway through AMPK

**Fig 5:**
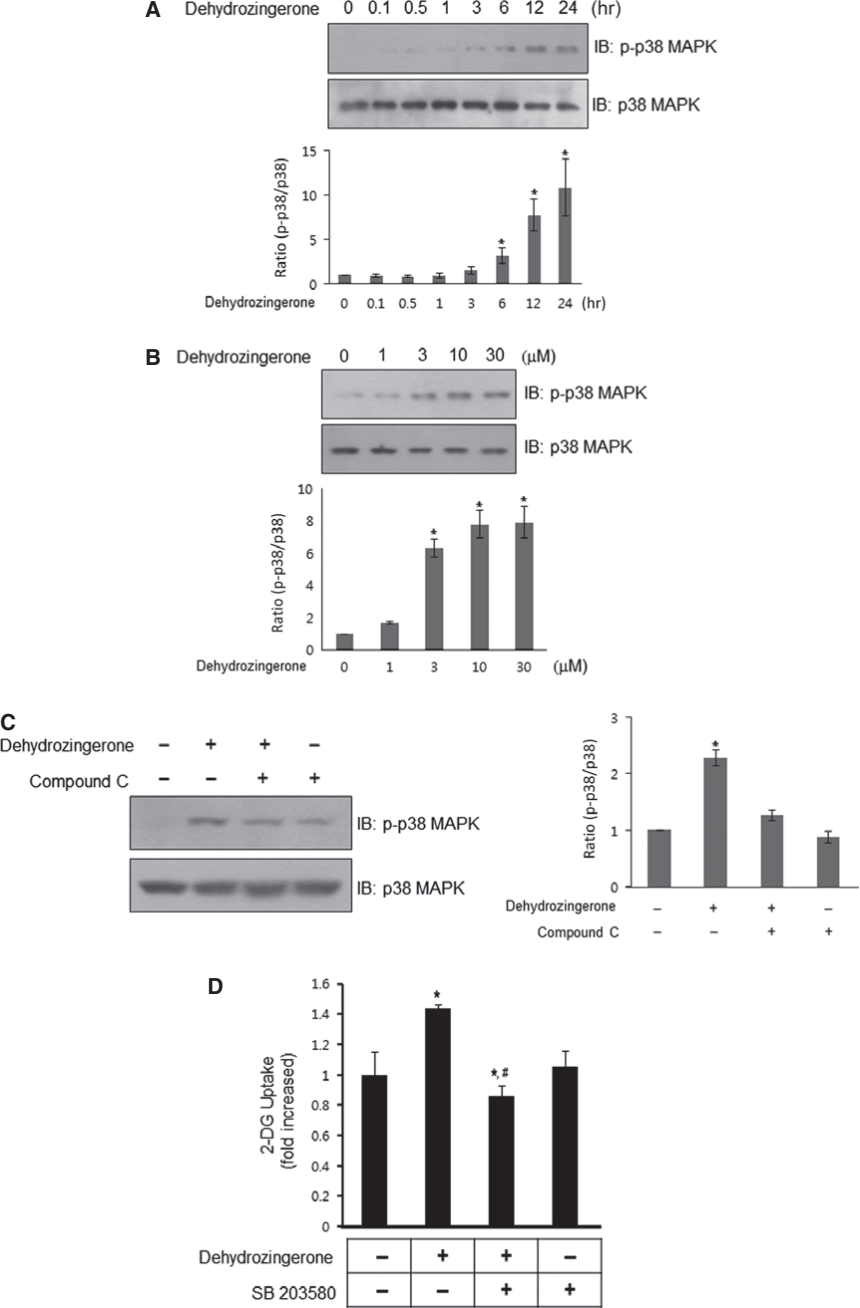
DHZ activates the p38 MAPK signal pathway. (A) C2C12 cells were stimulated with 30 μM DHZ for various times, as indicated. The cells were lysed with 2× SDS sample buffer, and the phosphorylation of p38 MAPK was evaluated by western blotting using phosphorylation-specific antibodies. The levels of total p38 MAPK were also assessed as a control for protein loading. The results are representative of four independent experiments. *P < 0.05 *versus* basal condition. (B) C2C12 cells were stimulated with various doses of DHZ. The cells were lysed with 2× SDS sample buffer, and the phosphorylation of p38 MAPK was evaluated by western blotting using phosphorylation-specific antibodies. The levels of total p38 MAPK were also assessed as a control for protein loading. The results are representative of four independent experiments. *P < 0.05 *versus* basal condition. (C) C2C12 cells were stimulated with 30 μM DHZ for 1 hr in the presence of compound C (2 μM). The cells were lysed with 2× SDS sample buffer, and the phosphorylation of p38 MAPK was evaluated by western blotting using phosphorylation-specific antibodies. The levels of total p38 MAPK were also assessed as a control for protein loading. Data are representative of four independent experiments. *P < 0.05 *versus* basal condition. (D) L6 myotubes were incubated with the p38 MAPK inhibitor SB203580 for 20 min., and cells were incubated for 1 hr in 12-well plates under the indicated conditions. 2-DOG uptake was then assayed. *P < 0.05, compared with control (one-way anova and post hoc Fisher's test); *^, #^*P* < 0.05, compared with DHZ-treated cells (one-way anova and Holm–Sidak comparisons). Each value is expressed as the mean ± SD of four independent experiments.

To better understand the signalling pathways involved in DHZ-induced glucose uptake, we investigated the effects of DHZ on p38 MAPK, a key molecule for glucose uptake 24. Treatment of C2C12 cells with 30 μM DHZ activated p38 MAPK in a time- and dose-dependent manner (Fig.5A and B). p38 MAPK plays a key role in AMPK-mediated glucose uptake 25. To assess the hierarchy of these effects, we measured p38 MAPK activation following the inhibition of AMPK with compound C. Treatment with compound C abrogated DHZ-induced p38 MAPK phosphorylation (Fig.5C). Next, we used the p38 MAPK inhibitor SB203580 to confirm the involvement of p38 MAPK on DHZ-induced glucose uptake. Inhibiting p38 MAPK also prevented DHZ-induced glucose uptake (Fig.5D). Together, these data suggest that p38 MAPK functions as a downstream of AMPK during DHZ-mediated glucose uptake.

### Effects of DHZ on GLUT4 expression

**Fig 6:**
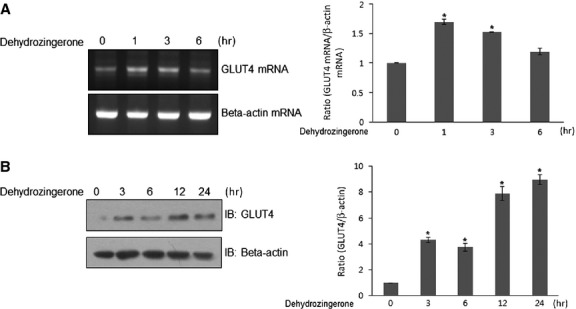
Effects of DHZ on GLUT4 expression. (A) Total mRNA was prepared from DHZ-treated cells, and RT-PCR was conducted using specific GLUT4 primers. The PCR products were then separated on 2% agarose gels and visualized under UV light. Beta-actin was used as a positive control. The results are representative of four independent experiments. *P < 0.05 *versus* basal condition. (B) C2C12 cells were stimulated for various times with DHZ. The cells were lysed with 2× SDS sample buffer, and the expression of GLUT4 was evaluated by western blotting. The levels of β-actin were also measured as a control for protein loading. The results are representative of four independent experiments. *P < 0.05 *versus* basal condition.

The principal glucose transporter that mediates glucose uptake is GLUT4, which plays a key role in regulating glucose homoeostasis. To assess the mechanism of DHZ-mediated glucose uptake, we examined the effect of DHZ on GLUT4 expression. Treatment with DHZ resulted in an increase in GLUT4 at both the mRNA and protein levels in C2C12 cells (Fig.6A and B). Localization of GLUT4 was not affected by DHZ (data not shown). These results suggest that DHZ could regulate glucose by inducing the expression of GLUT4.

### DHZ enhanced insulin-induced glucose uptake

**Fig 7:**
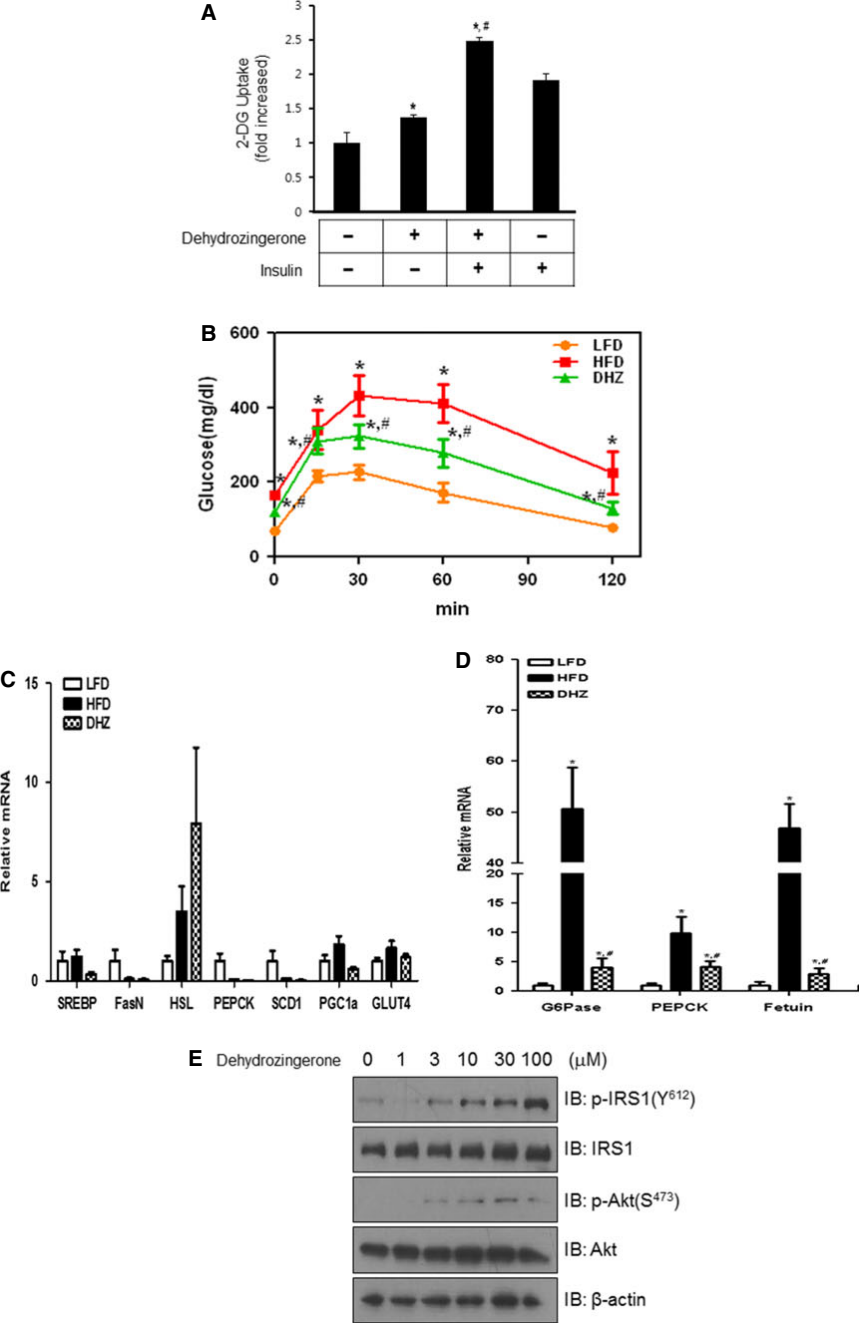
DHZ enhanced insulin-induced glucose uptake. (A) L6 myotubes were incubated with insulin in the absence or presence of DHZ for 1 hr in 12-well plates as indicated, and then 2-DOG was assayed. *P < 0.05, compared with control (one-way anova and post hoc Fisher's test); *^, #^*P* < 0.05, compared with DHZ-treated cells (one-way anova and Holm–Sidak comparisons). Each value is expressed as the mean ± SD of four independent experiments. (B) Effects of DHZ in a glucose tolerance test (GTT) in high-fat diet (HFD)-induced obesity model with the groups HFD or HFD supplemented with 100 mg/kg/day DHZ. Data are presented as means ± SD (*n* + 5). *Denotes significant differences compared with low-fat diet-fed mice (*P* < 0.05); *^, #^ denotes significant difference compared with HFD-fed mice (*P* < 0.05). (C) Total mRNA was prepared from the quadriceps muscle of each mouse, and RT-PCR was conducted using specific primers. The relative mRNA expression was analysed by qPCR (*n* + 5). (D) Total mRNA was prepared from the liver tissue of each mouse, and RT-PCR was conducted using specific primers. The relative mRNA expression was analysed by qPCR (*n* + 5). (E) C2C12 cells were stimulated with DHZ with the indicated doses. The cells were lysed with 2× SDS sample buffer, and the phosphorylation of IRS-1 and Akt was evaluated by western blotting using phosphorylation-specific antibodies. The levels of total IRS-1, Akt, and β-actin were also assessed as a control for protein loading. The results are representative of four independent experiments.

To confirm the effect of DHZ on glucose uptake, we assessed the effects of DHZ on insulin-stimulated glucose uptake. Co-treatment with DHZ potentiated insulin-induced glucose uptake (Fig.7A), suggesting that DHZ has an insulin sensitizing effect. If DHZ mediates beneficial effects on HFD-induced metabolic changes, increased activation of AMPK would lead to enhanced glucose clearance. To test this possibility, we performed an intraperitoneal glucose tolerance test in experimental animals using a 20% sucrose solution (2 g/kg). Blood glucose concentrations were significantly lower at 30 and 60 min. after glucose challenge in HFD + DHZ-fed mice than in HFD-fed mice, suggesting that DHZ-stimulated AMPK activation accelerated the clearance of blood glucose (Fig.7B). Next, we analysed tissues from skeletal muscle and liver. In skeletal muscles, most of the tested genes were not affected by either high-fat feeding or DHZ treatment (Fig.7C). One gene, hormone-sensitive lipase (HSL), increased in high-fat feeding and further increased by DHZ treatment. This result indicates that DHZ may affect HSL *via* DHZ-mediated hormonal changes. In liver tissues, up-regulation of two gluconeogenic genes, phosphoenolpyruvate carboxykinase (PEPCK) and glucose-6-phosphatase (G6Pase), was blocked by DHZ administration in high-fat feeding animal. The mRNA of fetuin, a marker of insulin resistance, was attenuated by DHZ (Fig.7D). To explore the molecular mechanisms underlying the effect of insulin sensitizing in skeletal muscle cell, we examined the effect of DHZ on IRS-1 and Akt. The phosphorylation IRS-1 and Akt increased by DHZ treatment in skeletal muscle cells (Fig.7E). Together, these findings suggest that DHZ exerts benign metabolic effects *via* insulin sensitivity in skeletal muscle and also *via* regulating gluconeogenic genes in liver.

## Discussion

The principal finding of our study was that DHZ, a structural analogue of curcumin, stimulates glucose uptake *via* the activation of AMPK in skeletal muscle. These findings suggest that the hypoglycaemic effects of curcumin are attributable to metabolic activity similar to that exerted by DHZ in skeletal muscles. The weight-lowering effect of DHZ was shown in normal feeding animal as well as high-fat feeding animal, probably because of its AMPK activation effect in tissues, such as skeletal muscle and adipocytes.

Curcumin regulates glucose homoeostasis in skeletal muscle *via* multiple mechanisms. A hypoglycaemic role for curcumin has been reported in a streptozocin-induced diabetic animal model 26. In addition, we previously reported that curcumin stimulated glucose uptake in skeletal muscle cells 22. Nevertheless, in spite of its clinical potential, curcumin has not yet been used as a therapeutic agent; this is predominantly because of poor water solubility 24 and low bioavailability 25 because of modest absorption. For example, negligible amounts of curcumin have been detected in the blood after oral administration of 1 g/kg curcumin 26. Bioavailability can also be decreased by metabolism. One study showed that 99% of curcumin in plasma existed as glucuronide conjugates 27. Several methods such as the use of adjuvants, nanoparticles and liposomes, have been suggested to overcome these limitations. The chemical structure of curcumin plays a critical role in its biological activity, and therefore structural modifications such as the use of analogues may enhance its solubility and bioavailability. In this study, we demonstrated that DHZ increased the phosphorylation of AMPK in skeletal muscle. Collectively, these findings suggest that a novel structural motif exists in curcumin structural analogues and that DHZ represents the primary motif that exerts effects on glucose metabolism. Liver is another organ for glucose regulation. In our data, DHZ treatment blocked the high-fat feeding induced change in gluconeogenic genes, such as G6Pase, PEPCK and insulin resistance marker, fetuin. These gluconeogenic genes and insulin resistance gene are induced in high-fat feeding animals, but dramatically suppressed by DHA co-treatment, indicating that metabolic roles of DHZ in liver may also contribute to phenotype changes of animal after DHZ administration.

It was recently reported that DHZ, a half analogue of curcumin, exhibits anti-oxidant, anti-tumour and lipid-peroxidation activities. Although only a few comparative studies have been performed on the effects of curcumin and DHZ on these parameters, structural variations may lead to changes in physiological activity. One study showed that the anti-oxidant effects of curcumin were more potent than those of DHZ. Curcumin has a number of molecular targets. However, because we did not compare their metabolic roles in skeletal muscle, we cannot rule out the possibility that DHZ was more effective at regulating glucose than curcumin. In the future, more extensive structure-function relationship studies should be performed. Another way to increase clinical utility is to develop structural analogues of DHZ, which may alter its pharmacokinetics to make it more easily absorbable in the intestine or more readily metabolizable to a more stable form. Curcuminoids are known to be poorly absorbed and rapidly metabolized *in vivo*. Curcuminoids undergo successive reduction to its metabolites in the liver by phase I metabolism, and also extensively conjugated with glucuronic acid by phase II metabolism. In case of curcumin, 65–85% of administrated doses was excreted unchanged in the faeces 26. One article showed that DHZ had similar brain uptake ability, but had lower accumulation in the liver, spleen and lungs 28. This result indicated that the instability of curcuminoids *in vivo* should not be ignored to evaluate its clinical usefulness.

Sulphonylureas and biguanides are drugs used to treat hyperglycaemia, but their use is limited by their accompanying side effects on the kidneys 29 and heart 30. Insulin therapy also does not guarantee a permanent normal pattern of glucose homoeostasis. Medicinal plants provide the advantage of exerting little or no side effects. To date, metformin, which originated from the plant *Galega officialis*, is the only plant-based drug approved for the treatment of diabetes. However, many medicinal plants might be useful sources for the development of potential diabetes drugs. In the field of diabetes, curcumin and its related analogues are increasingly recognized as potential novel therapeutics. In this study, we found that DHZ-stimulated glucose uptake *via* AMPK activation in skeletal muscle. The activation of p38 MAPK may also recruit specific signalling molecules for glucose uptake *via* AMPK activation. *In vivo*, DHZ blocked HFD-induced weight gain and increased blood glucose. In our data, DHZ activated AMPK-p38MAPK pathway in skeletal muscle cells and also exerted its benign roles in glucose homoeostasis. The association between p38 MAPK and glucose uptake was demonstrated in several articles 31–34, indicating that p38 MAPK activation in skeletal muscle cells might lead to benign effect in glucose regulation *in vivo*. In addition, some reports showed that p38MAPK caused brown adipocyte differentiation 35 and was involved in thermogenesis 36. These facts supported the notion that the activation of p38MAPK *in vitro* partially correlated with the bodyweight reduction in mice fed with DHZ. These findings provide a greater understanding of the hypoglycaemic functions of DHZ in skeletal muscle cells and suggest that it is a promising agent for the treatment of diabetes.
